# A multiaxial lead-free two-dimensional organic-inorganic perovskite ferroelectric

**DOI:** 10.1093/nsr/nwaa232

**Published:** 2020-09-08

**Authors:** Chao-Ran Huang, Xuzhong Luo, Xiao-Gang Chen, Xian-Jiang Song, Zhi-Xu Zhang, Ren-Gen Xiong

**Affiliations:** Key Laboratory of Organo-Pharmaceutical Chemistry of Jiangxi Province, College of Chemistry and Chemical Engineering, Gannan Normal University, Ganzhou 341000, China; Key Laboratory of Organo-Pharmaceutical Chemistry of Jiangxi Province, College of Chemistry and Chemical Engineering, Gannan Normal University, Ganzhou 341000, China; Jiangsu Key Laboratory for Science and Applications of Molecular Ferroelectrics, Southeast University, Nanjing 211189, China; Jiangsu Key Laboratory for Science and Applications of Molecular Ferroelectrics, Southeast University, Nanjing 211189, China; Jiangsu Key Laboratory for Science and Applications of Molecular Ferroelectrics, Southeast University, Nanjing 211189, China; Jiangsu Key Laboratory for Science and Applications of Molecular Ferroelectrics, Southeast University, Nanjing 211189, China

**Keywords:** ferroelectrics, organic-inorganic perovskites, multiaxial ferroelectrics, Curie temperature, thermochromism

## Abstract

Two-dimensional (2D) hybrid organic-inorganic perovskites (HOIPs) have recently gained tremendous interest because of their unique features in contrast to three-dimensional counterparts and traditional 2D materials. However, although some 2D HOIP ferroelectrics have been achieved, the issue of toxic Pb and uniaxial nature impede their further application. Herein, for the first time, we report a lead-free 2D HOIP multiaxial ferroelectric, [3,3-difluorocyclobutylammonium]_2_CuCl_4_ (**1**), which shows four ferroelectric axes and eight equivalent polarization directions, more than those of the other 2D HOIP ferroelectrics and even the inorganic perovskite ferroelectric BaTiO_3_ (three ferroelectric axes and six equivalent polarization directions). **1** also features a high Curie temperature of 380 K and exhibits remarkable thermochromism of color change from green-yellow to dark brown. To our knowledge, **1** is the first multiaxial lead-free 2D HOIP ferroelectric. This work sheds light on the exploration of better lead-free 2D HOIP ferroelectrics.

## Teaser

A lead-free two-dimensional organic-inorganic perovskite ferroelectric shows an unprecedented multiaxial feature with four ferroelectric axes and exhibits a high Curie temperature of 380 K.

## INTRODUCTION

Perovskite-type materials have received remarkable attention all over the world since they were discovered and investigated [1–3]. The large family of perovskites are acting as potential candidates for electronic and photoelectronic devices [4–6]. In recent years, the rapid development of three-dimensional (3D) CH_3_NH_3_PbI_3_-based perovskite solar cells has aroused great research interest in the area of low-cost and easily solution-processable hybrid organic-inorganic perovskites (HOIPs) [7–10]. Among them, the two-dimensional (2D) lead halide HOIPs, which have good moisture resistance, compositional diversity, quantum-well electronic structure, broadband emission and layer-tunable photoelectronic properties in contrast to 3D counterparts, are attracting increasing attention [11–14]. These features and the rich physical properties also make 2D HOIPs unique from conventional 2D materials including graphene, graphdiyne, MoS_2_ and WS_2_ [15,16]. 2D HOIPs adopt the typical crystal structure of organic cationic layer alternating with the 2D inorganic anionic framework [11–14]. This structural feature provides a great opportunity for arousing the ferroelectricity [17,18], which shows a crucial role in ferroelectric random access memory, piezoelectric devices and capacitors [19–23]. Ferroelectricity was found in a number of lead halide 2D HOIPs [24–35], such as (4,4-diflorocyclohexylammonium)_2_PbI_4_ [24], (C_5_H_10_CHNH_3_)_2_PbBr_4_ [25], (C_6_H_5_CH_2_NH_3_)_2_PbCl_4_ [26], [CH_3_(CH_2_)_3_NH_3_]_2_(CH_3_NH_3_)Pb_2_Br_7_ [27] and (C_4_H_9_NH_3_)_2_(NH_2_CHNH_2_)Pb_2_Br_7_ [28]. Some of them show excellent ferroelectric performance of high Curie temperature and large piezoelectric voltage coefficient [33,34]. However, the environmental toxicity of lead is an obstacle for their further applications. Within 2D HOIPs, the Pb(II) cation can be replaced by Sn(II), Ge(II), Cd(II), Mn(II), Fe(II), Cu(II) etc., while 2D HOIPs based on these metal cations are mainly investigated for the semiconducting property, photoluminescence, phase transitions and magnetism [36]. Recently, (C_4_H_9_NH_3_)_2_(NH_3_CH_3_)_2_Sn_3_Br_10_ was discovered as a potential ferroelectric without evidence of polarization switching [37], and Ye *et al*. reported 2D organic-inorganic double perovskite ferroelectrics [(*R*)- and (*S*)-3-hydroxylquinuclidinium]_4_KCe(NO_3_)_8_ [38]. Whereas, the lead-free 2D HOIP ferroelectrics remain very rare.

Regarding the application of ferroelectric materials, multiaxial ferroelectrics with multiple ferroelectric axes and equivalent polarization directions are highly preferable to uniaxial counterparts with one ferroelectric axis and two opposite polarization directions [39,40]. In the ferroelectric polycrystalline and thin-film sample, the more equivalent polarization directions allow easier and more effective polarization switching. Commercial ferroelectrics are generally multiaxial, and the multiaxial feature makes them widely used in the polycrystalline and thin-film form, as shown by the inorganic perovskite BaTiO_3_ with three ferroelectric axes and six equivalent polarization directions [39,40]. In this respect, however, most of the 2D HOIP ferroelectrics are only uniaxial or biaxial [24–35], which also greatly restricts their future application; for example, the lead-free [(*R*)- and (*S*)-3-hydroxylquinuclidinium]_4_KCe(NO_3_)_8_ are uniaxial ferroelectrics [38].

Here, we report a lead-free 2D Cu(II) halide HOIP multiaxial ferroelectric [3,3-difluorocyclobutylammonium]_2_CuCl_4_ ([DF-CBA]_2_CuCl_4_, **1**), which shows a 4/*mmm*F*m* ferroelectric phase transition at a high Curie temperature of 380 K, leading to four ferroelectric axes and eight equivalent polarization directions, larger than those of the other 2D HOIP ferroelectrics and the BaTiO_3_. Interestingly, **1** also presents prominent thermochromic behavior of color change from green-yellow to dark brown, which is not observed in lead halide 2D HOIP ferroelectrics. To our knowledge, **1** is the first multiaxial lead-free 2D HOIP ferroelectric. This pioneering work throws light on the exploration of new, excellent multiaxial lead-free 2D HOIP ferroelectrics with application prospects.

## RESULTS AND DISCUSSION

**Figure 1.**
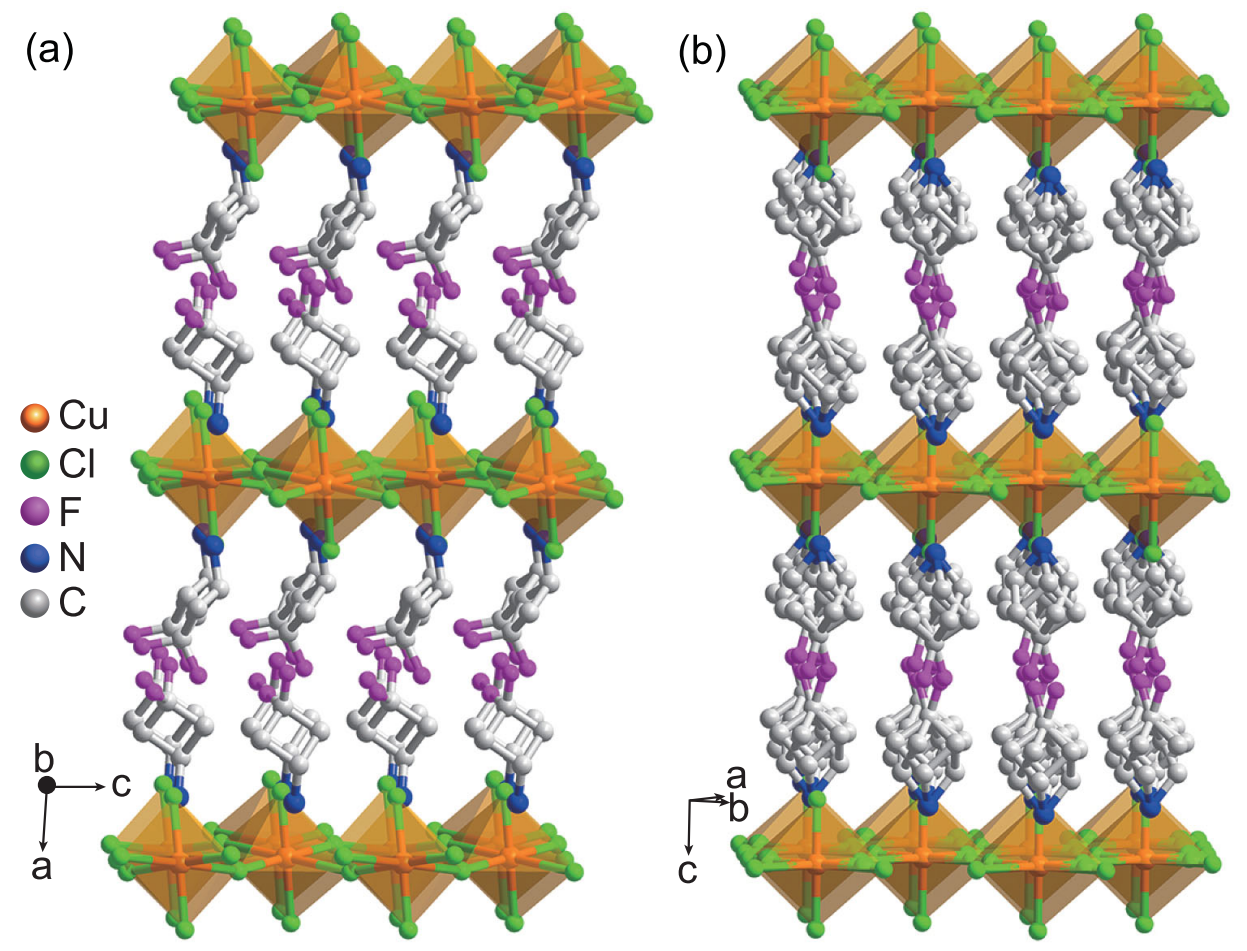
Packing views of the crystal structures of **1** at (a) 293 K and (b) 393 K.

We obtained single-crystals of **1** by slow evaporation of a methanol solution containing [3,3-difluorocyclobutylammonium]Cl and CuCl_2_. Single-crystal X-ray diffraction experiments reveal that **1** crystallizes in the monoclinic polar *Cc* space group (point group *m* (*C*_1h_)) at 293 K in the ferroelectric phase (FP) (Supplementary Table S1). The crystal structure contains inorganic [CuCl_4_]^2−^ layers of corner-sharing CuCl_6_ octahedral separated by organic [DF-CBA]^+^ bilayers (Fig. 1a), showing the 2D organic-inorganic A_2_BX_4_ perovskite structure [36], where the [DF-CBA]^+^, Cu^2+^ and Cl^−^ ion corresponds to the A, B and X site, respectively. Thus, **1** can be regarded as the 2D halide perovskite, similar to the case of [CH_3_NH_3_]_2_CuCl_4_ [41]. The CuCl_6_ octahedron is Jahn-Teller distorted with two Cu–Cl bond lengths (3.1635 and 3.0523 Å) obviously longer than the other four (2.2583–2.3179 Å) (Supplementary Table S2). The alternate elongated Cu–Cl bonds of the Jahn-Teller distorted CuCl_6_ octahedra are perpendicular to each other, showing an anti-ferrodistortive arrangement in the *bc* plane (Supplementary Fig. S1), as found in [2,2′-(ethylenedioxy)bis(ethylammonium)]CuCl_4_ [42]. The [DF-CBA]^+^ cations are orientationally ordered, of which the C−N bonds align along one single orientation (Fig. 1a). The polar [DF-CBA]^+^ cation with electric dipole moment shows an orientational arrangement along the *c* axis with all the C–N groups of [DF-CBA]^+^ cations aligning along the *c* axis (Supplementary Fig. S2). Such an orientational arrangement of polar cations will induce a polarization [26]. Between the inorganic and organic components, there are weak N–H···Cl hydrogen-bonding interactions (Supplementary Fig. S3a), in which the average donor-acceptor distance is 3.350 Å.

At 393 K in the paraelectric phase (PP), the crystal symmetry of **1** changes to the tetragonal centrosymmetric *P*4_2_/*mmc* space group (point group 4/*mmm* (*D*_4h_)). The relationship between the unit cell of FP and PP is *a*^FP^ ≈ *c*^PP^, *b*^FP^ ≈ $\sqrt 2 $*a*^pp^ and *c*^FP^ ≈ $\sqrt 2 $*b*^pp^. In the crystal structure, both the inorganic and organic constituents get seriously disordered (Fig. 1b). The atomic coordinates of the four bridged Cl atoms of CuCl_6_ octahedron were split into two positions (Fig. 1b and Supplementary Fig. S3b). The [DF-CBA]^+^ cation exhibits a 4-fold orientational disorder with the C−N bonds having four orientations because it occupies the 2*mm* symmetry positions (Fig. 1b and Supplementary Fig. S3b). Thus, both the order-disorder transitions of the inorganic [CuCl_4_]^2−^ framework and the organic [DF-CBA]^+^ cations contribute to the ferroelectric-to-paraelectric phase transition. In PP, the dipole moments of [DF-CBA]^+^ cations cancel each other out because of the centrosymmetric crystal symmetry. We also confirm the structural phase transition of **1** by the variable-temperature powder X-ray diffraction (PXRD) experiments (Supplementary Fig. S4). The PXRD patterns at 293 K are consistent with the simulated ones from the crystal structure (Supplementary Fig. S4b) and show no obvious change with temperature increasing in FP. When the temperature increases to PP, the numbers of PXRD patterns reduce obviously, especially in the 2*θ* range from 16° to 24° (Supplementary Fig. S4a), which indicates a higher symmetry in PP. The experimental PXRD patterns at 393 K also match the simulated ones from the crystal structure (Supplementary Fig. S4c).

**Figure 2.**
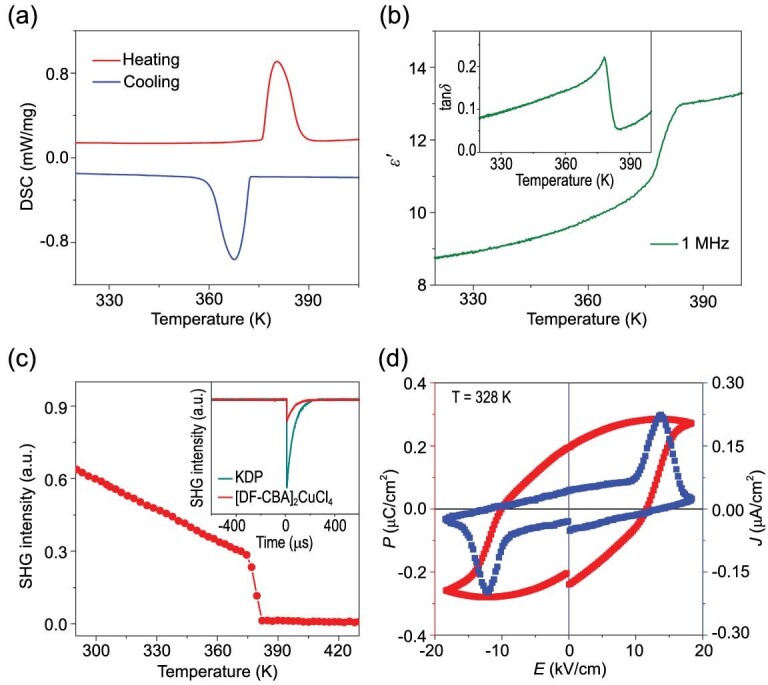
Ferroelectricity and related properties of **1**. (a) DSC curves. (b) Temperature-dependent *ϵ*′ at 1 MHz upon heating. Inset: temperature-dependent tan*δ* at 1 MHz upon heating. (c) Temperature-dependent SHG intensity upon heating. Inset: SHG signals of KDP and **1** at 293 K. (d) Ferroelectric hysteresis loop measured on the single-crystal sample along the *c* axis at 328 K by using the double-wave method.

**Figure 3.**
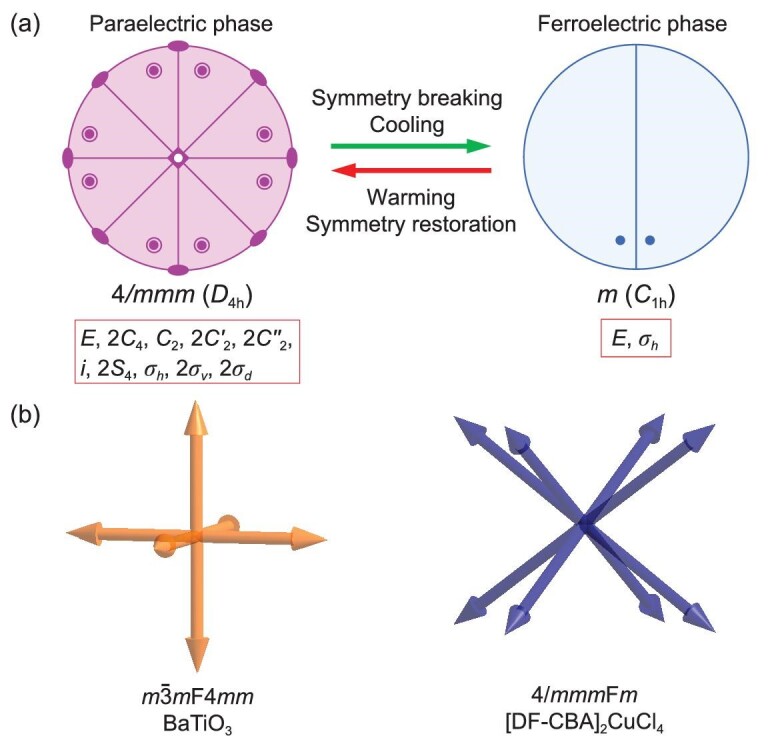
(a) Symmetry change between the PP and FP in **1**. (b) Comparison of the polarization directions in BaTiO_3_ and **1**.

Differential scanning calorimetry (DSC) experiments reveal that the ferroelectric-to-paraelectric phase transition of **1** occurs at *T*_c_ = 380 K (*T*_c_ is the phase transition temperature) (Fig. 2a). The phase transition is reversible and repeatable as reflected in the DSC curves recorded in three heating/cooling cycles (Supplementary Fig. S5). Thermogravimetric analysis (TGA) curves show that **1** is thermally stable up to 480 K (Supplementary Fig. S6), which is much higher than the phase transition temperature of 380 K. The dielectric anomaly near *T*_c_ in the temperature-dependent real part (*ϵ*′), loss (tan*δ*), and imaginary part (*ϵ*′′ = *ϵ*′ × tan*δ*) of the complex permittivity of **1** at 1 MHz further verifies this phase transition (Fig. 2b and inset, and Supplementary Fig. S7). The *ϵ*′ at 1 kHz, 10 kHz and 100 kHz shows obvious dielectric anomalies near 380 K as well (Supplementary Fig. S8). At lower frequencies, the dielectric anomalies are remarkable and become more like a peak shape (Supplementary Fig. S8). The peak temperature of the dielectric anomalies shows no obvious shift from the frequency of 1 kHz to 1 MHz, showing no dielectric relaxations. We also employed the temperature-dependent second harmonic generation (SHG) signal to study the phase transition. The SHG intensity of **1** has a certain value of about a quarter of that of KDP (potassium dihydrogen phosphate) at 293 K in the FP. It decreases to a zero value near *T*_c_, and maintains zero values in the PP. This corresponds to the transition from the polar point group *m* (*C*_1h_) to a centrosymmetric 4/*mmm* (*D*_4h_) one. According to Aizu notion [43], the phase transition in **1** is the 4/*mmm*F*m* type ferroelectric. As Fig. 3a shows, from PP to FP, the symmetry breaks from 16 symmetry elements (*E*, 2*C*_4_, *C*_2_, 2*C*′_2_, 2*C*′′_2_, *i*, 2*S*_4_, *σ_h_*, 2*σ_v_* and 2*σ_d_*) in the 4/*mmm* (*D*_4h_) point group to two (*E* and *σ_h_*) in the *m* (*C*_1h_) point group. Thus, **1** is a multiaxial ferroelectric with four ferroelectric axes and eight equivalent polarization directions. It is noted that the polarization directions of **1** are much more than those of other 2D HOIPs, most of which are uniaxial or biaxial ferroelectrics [24–35], and even more than the six polarization directions of typical inorganic perovskite ferroelectric BaTiO_3_ (Fig. 3b) [39,40]. The multiaxial nature of **1** facilitates its applications in the thin-film form [39,40].

We then recorded the polarization-electric field (*P*–*E*) hysteresis loop of **1**. As shown in Fig. 2d, **1** presents a *P*–*E* hysteresis loop obtained on the single-crystal sample along the *c* axis at 328 K in the FP, directly proving the ferroelectricity. The saturate polarization (*P*_s_) is estimated to be 0.29 *μ*C/cm^2^ from the loop, which is larger than that of the first molecular ferroelectric Rochelle Salt (0.25 *μ*C/cm^2^) [44]. We also calculated the *P*_s_ of **1** based on the point charge mode (Supplementary Fig. S9). The experimental value is comparable to the calculated one (0.328 *μ*C/cm^2^).

**Figure 4.**
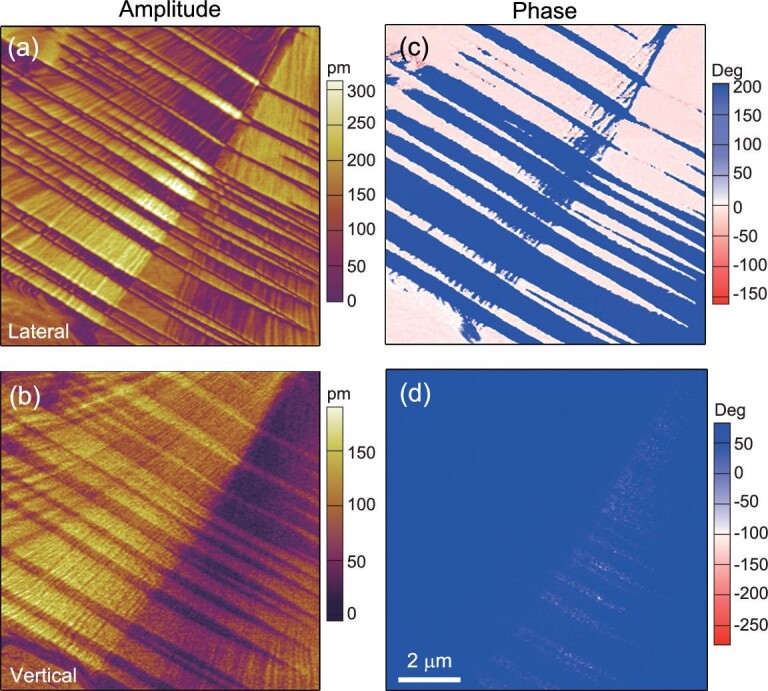
PFM imaging of domain pattern in the thin film of **1.** Lateral (a) and vertical (b) PFM amplitude images. Lateral (c) and vertical (d) PFM phase images.

We also characterized the domain structure of **1** on its thin film by piezoresponse force microscopy (PFM). The typical domain pattern was arranged in a stripe texture. An example is shown in Fig. 4. The stripe contrast can be clearly observed in both lateral and vertical amplitude images, in which the light and dark tones represent the local piezoresponse along the in-plane direction for lateral image and out-of-plane direction for vertical image. By comparison of the phase images, a uniform phase in the vertical phase image is seen. Contrastingly, in the lateral phase image the stripe domains were observed. The phase anisotropy of the vertical and lateral PFM modes indicates the existence of non-180° domain walls in the thin film, which thus reveals that **1** is a multiaxial ferroelectric.

**Figure 5.**
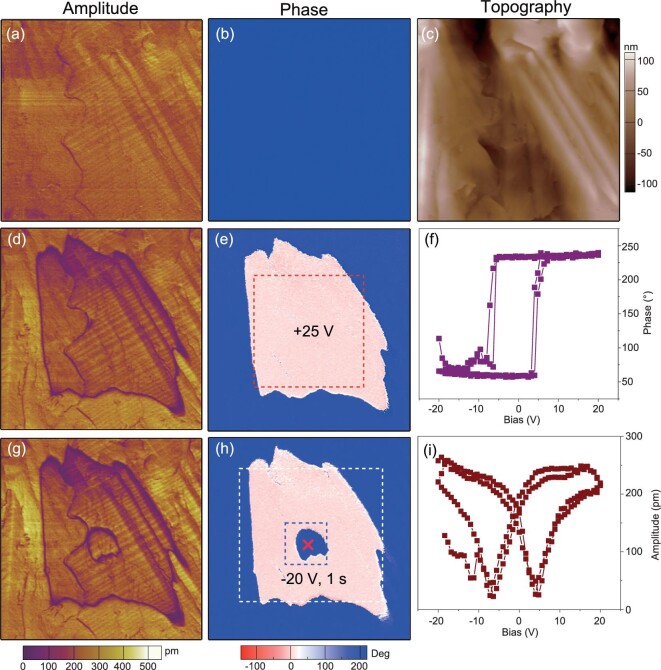
Polarization switching experiments for the thin film of **1.** (a) Initial PFM amplitude, (b) PFM phase and (c) topographic images of a region of 20 × 20 μm. (d) PFM amplitude and (e) phase images after applying voltage of +25 V on the red-boxed region. (g) PFM amplitude and (h) phase images after applying −20 V voltage pulse of 1 s duration on the point ×. PFM switching spectroscopy showing (f) square-like phase loops and (i) butterfly-like amplitude loops.

Figure 5a and b respectively display the pristine vertical PFM amplitude and phase images of a region of 20 × 20 μm, together with the surface topography image shown in Fig. 5c. By locally poling the red-boxed region marked in Fig. 5e with positive voltage of +25 V, we found that a new domain was generated through the polarization switching. Next, a –20 V, 1 s duration voltage pulse was applied at the position ‘×’ in the new domain. As a result, the partial of the new domain was switched back with 180° phase shift. Furthermore, local ferroelectric hysteresis loops can also be recorded by switching spectroscopy PFM measurements. A set of direct current (DC) voltage pulses up to ±20 V were applied to switching the polarization underneath the PFM tip, with a 5 V ac tip bias applied to record the corresponding piezoelectric amplitude and phase signal. As shown in Fig. 5f, 180° phase reversal occurs when a coercive voltage is exceeded. At the same time, hysteresis amplitude-bias butterfly loops were observed (Fig. 5i). Overall, our PFM measurements provide robust proof of the switchable polarization in **1**.

**Figure 6.**
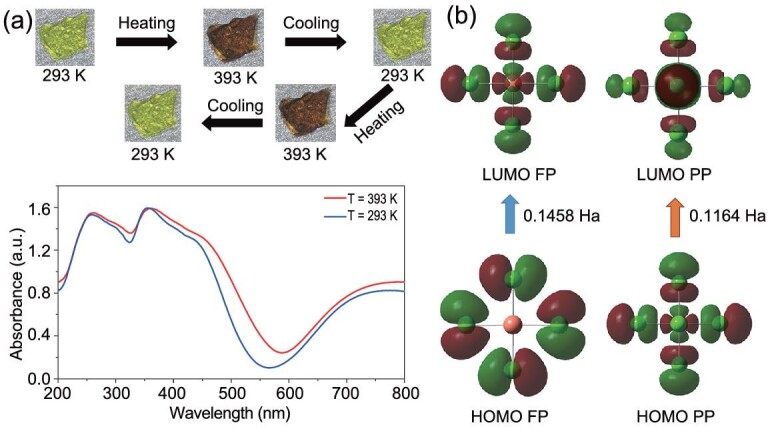
(a) Crystal (size: 1.1 × 0.9 × 0.5 mm^3^) color and solid-state UV–Vis absorption spectra of **1** at 293 and 393 K. (b) The HOMO and LUMO of CuCl_6_ octahedral configurations in FP and PP of **1**.

Interestingly, distinct from the 2D lead halide perovskite ferroelectrics, **1** exhibits thermochromic behaviors. Its crystal changes color obviously, reversibly and repeatedly from yellow-green at 293 K to a dark brown color at 393 K (Fig. 6a). The ultraviolet-visible (UV–Vis) absorption spectra at 293 K shows that **1** mainly absorbs light less than about 530 nm, matching with its yellow-green appearance. It is worth noting that there is a minor and broad absorption peak at around 750 nm in the absorption spectra, which is commonly found in 2D copper (II) chloride HOIP perovskites like [CH_3_NH_3_]_2_CuCl_4_ [41], and this absorption peak can be assigned to the *d*–*d* electronic transitions in the Cu^2+^ ions with the 3*d*^9^ electronic configuration [41]. When the temperature increases, the main absorption band in the UV–Vis absorption spectra shows a remarkable red shift from 293 to 393 K (Fig. 6a). We also recorded the UV–Vis absorption spectra at different temperatures (Supplementary Fig. S10). The main absorption peaks at around 257 and 354 nm at 293 K show very slight red shifts when the temperature increases to 393 K. For the main absorption peak at around 435 nm at 293 K, it presents minor red shifts with temperature increasing below *T*_c_ of 380 K, and shows a relatively remarkable red shift from 373 K below the *T*_c_ to 393 K above *T*_c_. When the temperature increases, the absorption edges of the main visible absorption band at around 524 nm at 293 K also show a remarkable red shift from 373 to 393 K. This indicates that the thermochromism of **1** is associated with the phase transition, as found in other thermochromic organic-inorganic Cu(II) halides showing phase transitions, in which the thermochromism is mainly related to the coordination environment change of copper (II) ion during the phase transition [45–47]. In **1**, the out-plane Cl–Cu–Cl bond angles of 86.43°–94.43° at 293 K in FP become 90.00° at 393 K in PP (Supplementary Fig. S11 and Table S2). The average long in-plane Cu–Cl bond distance of 3.1515 Å, the average short in-plane Cu–Cl bond distance of 2.2590 Å and the average out-plane Cu–Cl bond distance of 2.2321 Å in PP are also obviously different from those in FP (3.1075 Å, 2.3155 Å and 2.2592 Å respectively). The changes of Cl–Cu–Cl bond angles and Cu–Cl bond distance show the geometry change of CuCl_6_ octahedron, which contributes to the thermochromism.

In order to investigate the underlying cause of thermochromism, the HOMO (highest occupied molecular orbital) and LUMO (lowest unoccupied molecular orbital) of CuCl_6_ octahedral configurations in FP and PP are calculated (Fig. 6b). The HOMO and LUMO in PP display distinct variation from those in FP. The resultant energy gap shifted from 0.1458 Ha (Hartree) in the FP to 0.1164 Ha of PP. The large energy gap change could be attributed to the remarkable configuration change of CuCl_6_ octahedral. Accordingly, the absorption edge will have a red shift tendency in the heating process from FP to PP, which is consistent with the experimental variable-temperature UV–vis absorption spectra.

## CONCLUSION

In summary, we have presented a lead-free 2D HOIP ferroelectric, [3,3-difluorocyclobutylammonium]_2_CuCl_4_, which undergoes a 4/*mmm*F*m* type ferroelectric phase transition at 380 K. Strikingly, **1** is a multiaxial ferroelectric with eight polarization directions, much more than those of 2D lead halide perovskite ferroelectrics, facilitating its application in the thin-film form. This is the first example of a 2D lead-free perovskite multiaxial ferroelectric. Moreover, during the ferroelectric-to-paraelectric phase transition, **1** shows remarkable thermochromism of color change from green-yellow to dark brown. This work opens up a pathway to constructing multiaxial lead-free 2D perovskite ferroelectrics.

## METHODS

### Sample preparation

The [3,3-difluorocyclobutylammonium]Cl ([DF-CBA]Cl) and CuCl_2_ are commercially available. [DF-CBA]Cl (10 mmol) and CuCl_2_ (5 mmol) were completely dissolved in methanol (100 ml). Slow evaporation of the solvent at room temperature obtains the crystals of [DF-CBA]_2_CuCl_4_ (yield, 98.5%). Single-crystal X-ray diffraction experiments reveal that the obtained crystals have the formula of [DF-CBA]_2_CuCl_4_ (Supplementary Table S1). The phase purity of the as-grown crystals was confirmed by PXRD (Supplementary Fig. S4b). We prepared the thin film of [DF-CBA]_2_CuCl_4_ by spreading 10 μL precursor solution (100 mg as grow-crystals in 1 mL methanol) on ITO (indium tin oxide) coated glass substrate. Annealing the substrate at 313 K obtains the thin-film sample.

### Characterization methods

Methods of XRD, DSC, dielectric, SHG, *P*–*E* loop, PFM and UV–vis absorption spectra measurements were described in detail previously [26,32]. The HOMO and LUMO are calculated at b3lyp/aug-cc-pVTZ level.

### Accession codes

The structures have been deposited at the Cambridge Crystallographic Data Centre (deposition numbers: CCDC 1982768–1982769).

## Supplementary Material

nwaa232_Supplemental_File
