# Supplementary material for: A multiaxial lead-free two-dimensional organic-inorganic perovskite ferroelectric
Source: Natl Sci Rev. 2020 Sep 8;8(5):nwaa232. doi: 10.1093/nsr/nwaa232 (PMC8288432; doi:10.1093/nsr/nwaa232)
Supplement: nwaa232_Supplemental_File [file nwaa232_supplemental_file.docx]

**Supplementary Information**

**A Multiaxial Lead-free Two-Dimensional Organic-Inorganic Perovskite Ferroelectric**

Chao-Ran Huang^1^, Xuzhong Luo^1,^*, Xiao-Gang Chen^2^, Xian-Jiang Song^2^, Zhi-Xu Zhang^2^ and Ren-Gen Xiong^2,^*

^1^Key Laboratory of Organo-Phamaceutical Chemistry of Jiangxi Province, College of Chemistry and Chemical Engineering, Gannan Normal University, Ganzhou 341000, China

^2^Jiangsu Key Laboratory for Science and Applications of Molecular Ferroelectrics, Southeast University, Nanjing 211189, China

***Corresponding authors**. E-mails: luoxz@gnnu.edu.cn; xiongrg@seu.edu.cn


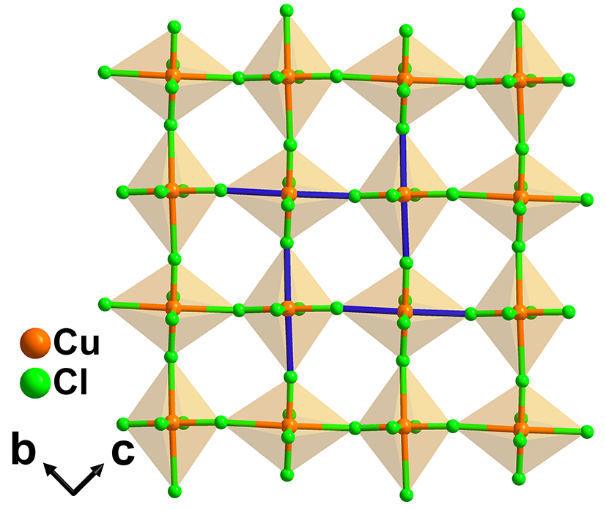


**Figure S1.** A view of the 2D [CuCl_4_]^2-^ layer, showing an anti-ferrodistortive arrangement of the Jahn-Teller distorted CuCl_6_ octahedra in the *bc* plane. Selected elongated Cu–Cl bonds are marked in blue color for clarity, which are perpendicular to each other.


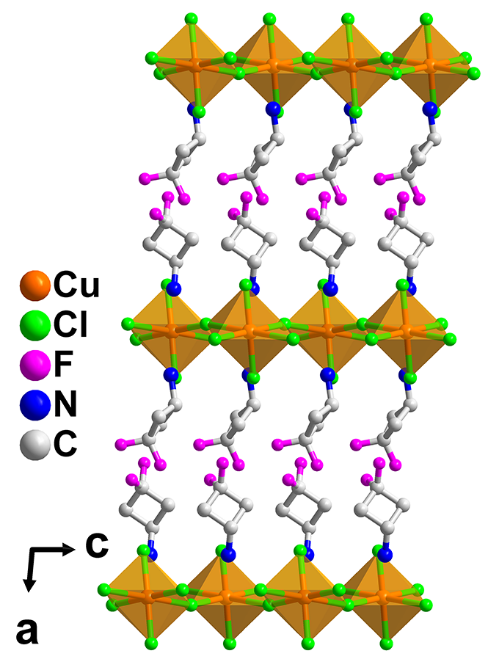


**Figure S2.** Packing view of crystal structure of [DF-CBA]_2_CuCl_4_ at 293 K in FP along the *b* axis. The polar [DF-CBA]^+^ cations show an orientational arrangement along the *c* axis with all the C–N groups of [DF-CBA]^+^ cations aligning along the *c* axis.


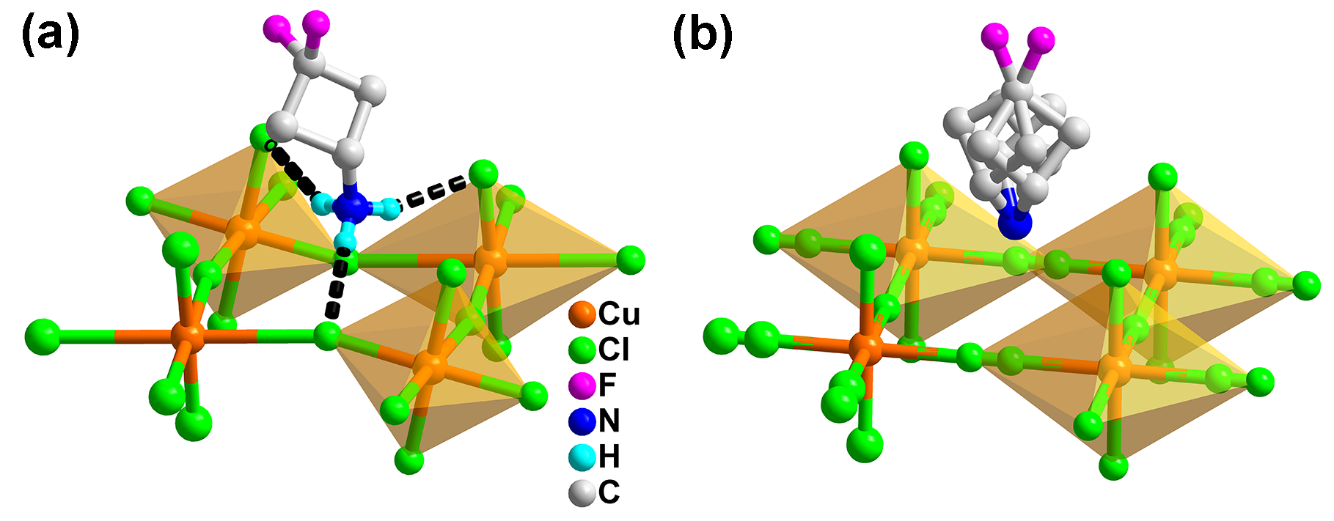


**Figure S3.** Molecular structure of [DF-CBA]_2_CuCl_4_ at (a) 293 K and (b) 393 K.


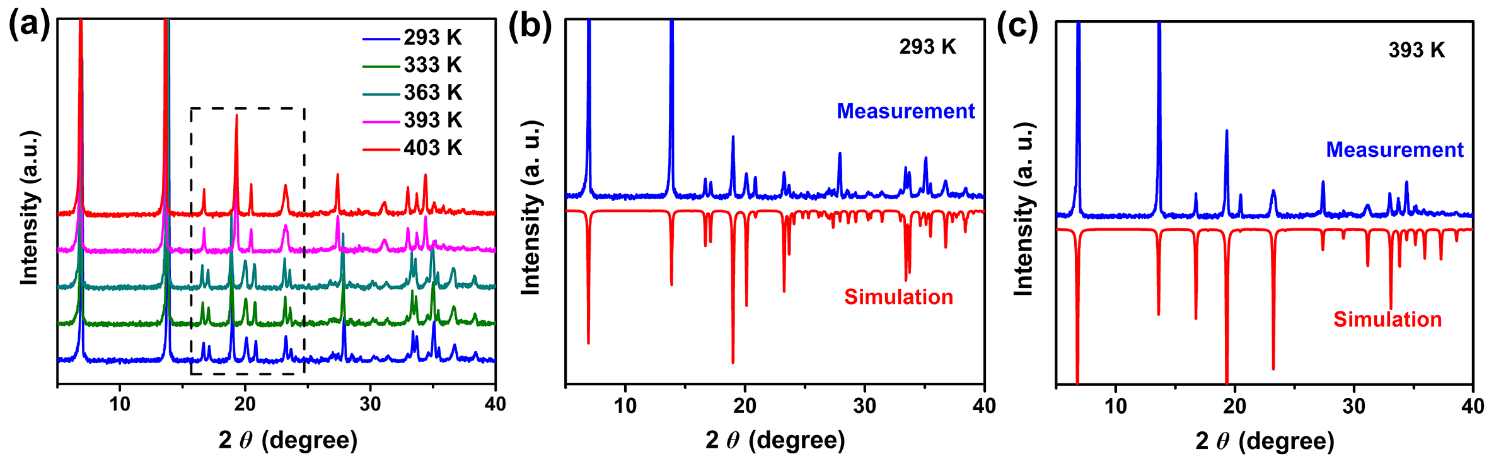


**Figure S4.** (a) Variable-temperature PXRD patterns of [DF-CBA]_2_CuCl_4_. Experimental PXRD patterns [DF-CBA]_2_CuCl_4_ at (b) 293 K and (c) 393 K matching with the simulated ones from crystal structures at 293 K and 393 K, respectively.


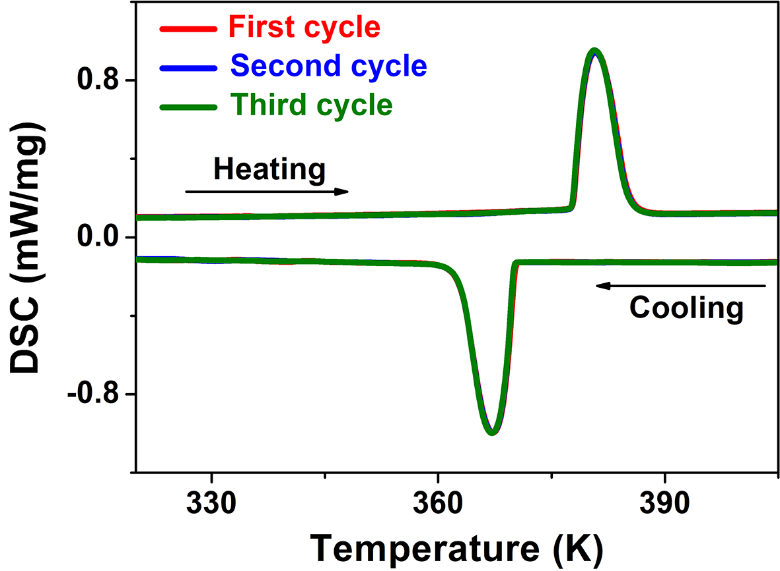


**Figure S5.** DSC curves recorded in three heating/cooling cycles.


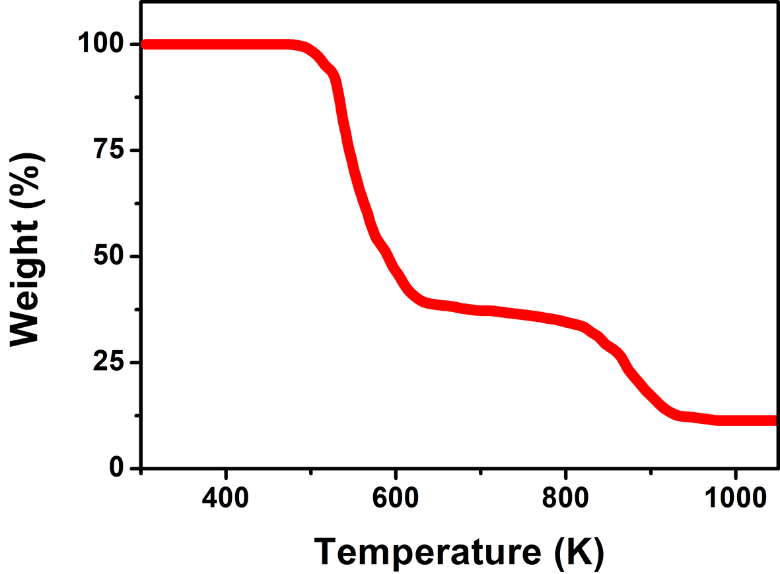


**Figure S6.** Thermogravimetric analysis (TGA) curve of [DF-CBA]_2_CuCl_4_.


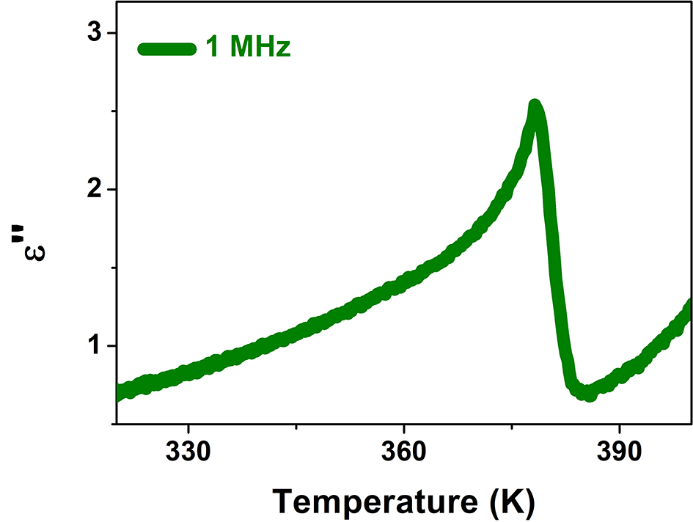


**Figure S7.** Temperature-dependent *ε*′′ at 1 MHz upon heating.


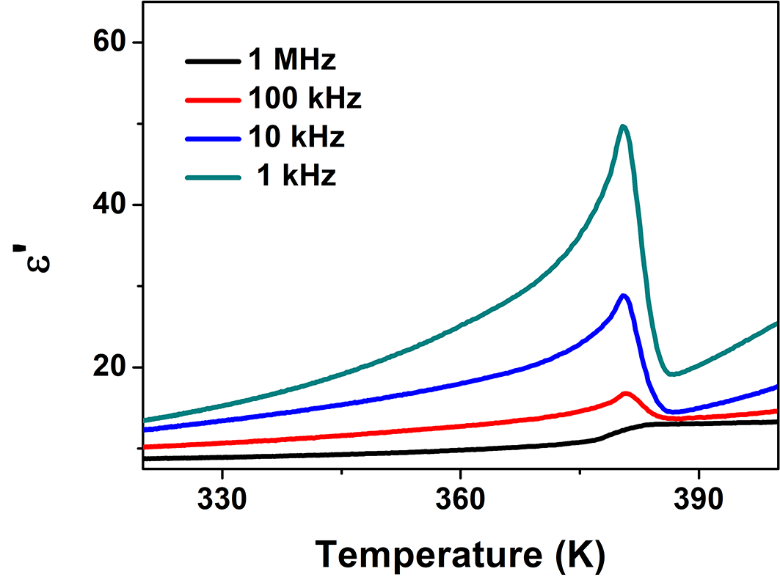


**Figure S8.** Temperature-dependent *ε*′ at selected frequencies upon heating.


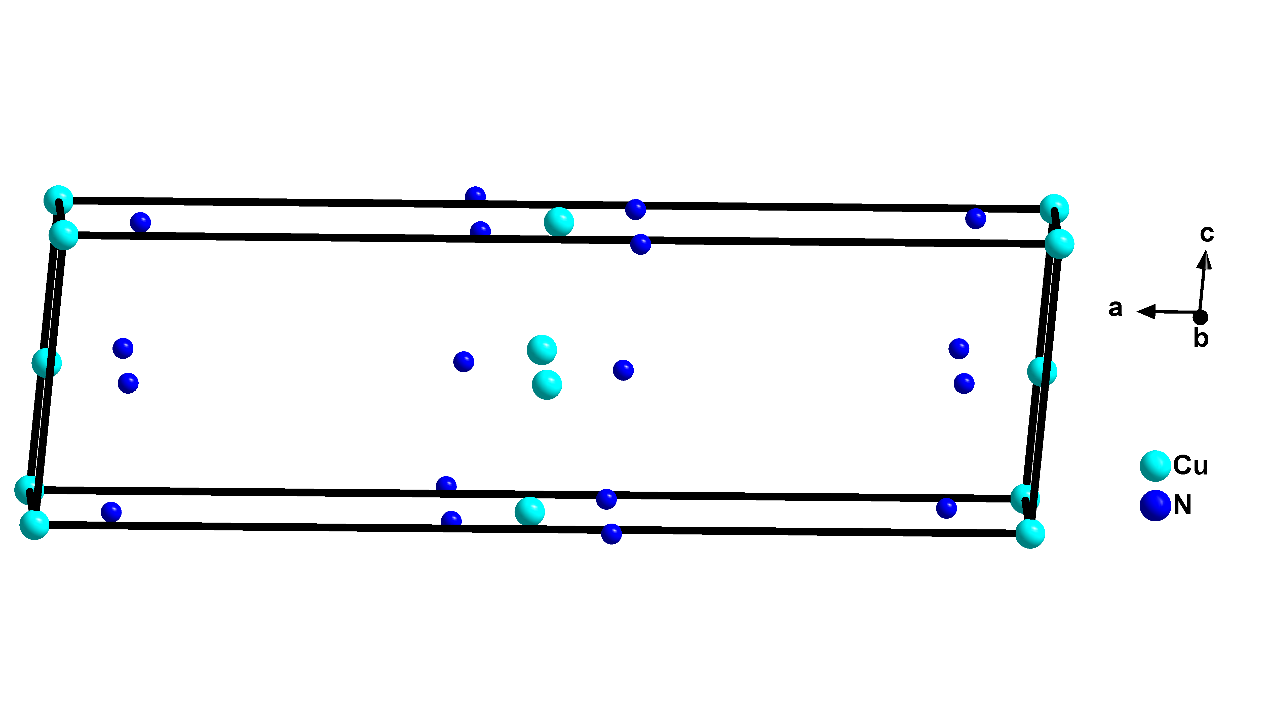


**Figure S9.** Distribution of N and Cu atoms of [DF-CBA]_2_CuCl_4_ in the reselected equivalent ferroelectric phase unit cell at 293 K.

**Point Charge Calculation on Saturated Polarization**

According to the crystal structure data collected at 293 K, we select a unit cell and assume that the center of the positive charge locates on N atoms, and negative charges on Cu atoms, respectively.

| Atoms | Center Coordination |
| --- | --- |
| Cu | (0.16583, 0.25000, 0.32915) |
| N | (0.16690, 0.25000, 0.33415) |

*P_s//c_­­ =* lim$\frac{1}{V}\sum q_{i}r_{i}$

= (*q*_Cd_*r*_Cd_ + *q*_N_*r*_N_) / V

= [(−2×4×e×0.32915)+ (8×e×0.33415)]×c/V

= −[0.005×8×1.6 × 10^−19^ ×7.5407 × 10^−10^ C m] / (1472.1× 10^−30^ m^3^)

= −3.28×10^−3^C m^−2^

|*P_s//c_|­* = 0.328 *μ*C cm^−2^


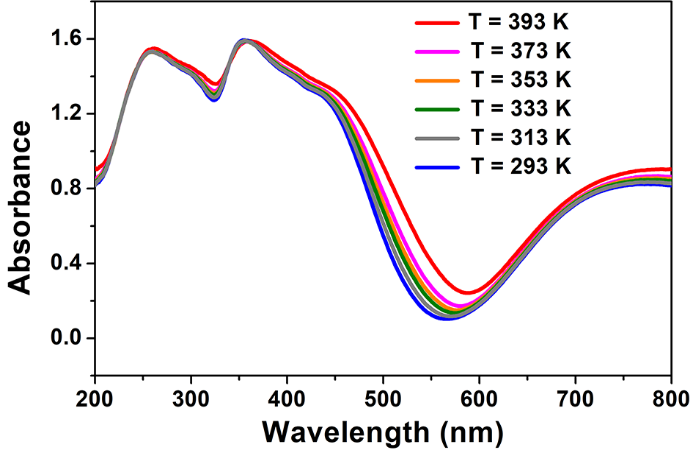


**Figure S10.** Solid-state UV-Vis absorption spectra of [DF-CBA]_2_CuCl_4_ at different temperatures.


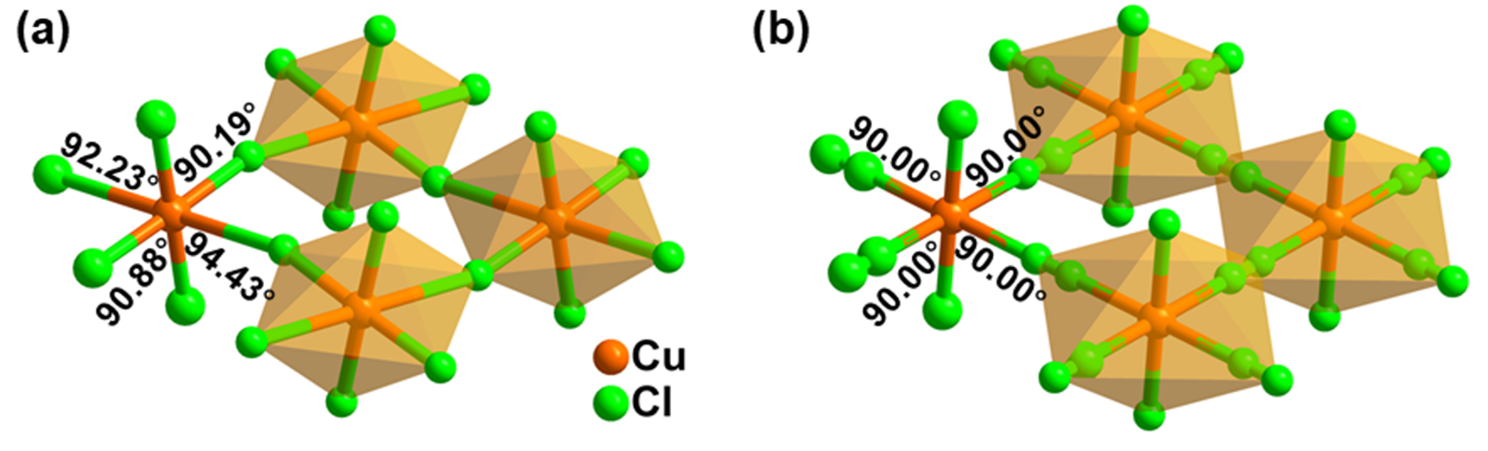


**Figure S11.** Comparison of the CuCl_6_ octahedron of inorganic [CuCl_4_]^2-^ framework in [DF-CBA]_2_CuCl_4_ at (a) 293 K and (b) 393 K with selected Cl–Cu–Cl bond angles labled.

**Table S1.** Crystal data and structure refinements for [DF-CBA]_2_CuCl_4_ at 293 K and 393 K, respectively.

| Formula | [F_2_C_4_H_7_NH_3_]_2_CuCl_4_ | |
| --- | --- | --- |
| Temperature | 293 K | 393 K |
| Weight | 421.58 | 421.58 |
| Crystal system | Monoclinic | Tetragonal |
| Space group | *Cc* | *P*4_2_/*mmc* |
| *a*/Å | 25.645 (3) | 5.4107 (4) |
| *b*/Å | 7.6429 (7) | 5.4107 (4) |
| *c*/Å | 7.5407 (7) | 26.045 (5) |
| *β*/deg | 95.099 (9) | 90 |
| Volume/Å^3^ | 1472.1 (2) | 762.49 (18) |
| *Z* | 4 | 2 |
| *R*1 [ *I>2σ*(*I*)] | 0.0460 | 0.1531 |
| *wR*2 [ *I>2σ*(*I*)] | 0.1217 | 0.3349 |
| GOF | 1.001 | 1.006 |

**Table S2.** Selected Cl−Cu bond lengths [Å] and Cl−Cu−Cl bond angles [°] for [DF-CBA]_2_CuCl_4_ at 293 K.

| Temperature | bond lengths [Å] | | bond angles [°] | |
| --- | --- | --- | --- | --- |
| **293 K** | Cu1—Cl2 | 2.2583 (26) | Cl2—Cu1—Cl4 | 178.67 (11) |
|  | Cu1—Cl4 | 2.2601 (26) | Cl2—Cu1—Cl1 | 90.88 (10) |
|  | Cu1—Cl1 | 2.3129 (23) | Cl4—Cu1—Cl1 | 89.17 (10) |
|  | Cu1—Cl3 | 2.3179 (26) | Cl2—Cu1—Cl3 | 89.74 (10) |
|  | Cu1—Cl1^i^ | 3.1635 (23) | Cl4—Cu1—Cl3 | 90.19 (9) |
|  | Cu1—Cl3^ⅱ^ | 3.0523 (26) | Cl1—Cu1—Cl3 | 178.75 (9) |
|  |  |  | Cl1^i^—Cu1—Cl1 | 87.83 (7) |
|  |  |  | Cl1^i^—Cu1—Cl2 | 86.43 (9) |
|  |  |  | Cl1^i^—Cu1—Cl3 | 91.13 (10) |
|  |  |  | Cl1^i^—Cu1—Cl4 | 92.23 (9) |
|  |  |  | Cl1—Cu1—Cl3^ⅱ^ | 91.93 (7) |
|  |  |  | Cl2—Cu1—Cl3^ⅱ^ | 94.43 (9) |
|  |  |  | Cl3—Cu1—Cl3^ⅱ^ | 89.91 (8) |
|  |  |  | Cl4—Cu1—Cl3^ⅱ^ | 86.90 (9) |
|  |  |  | Cu1—Cl1—Cu1^ⅲ^ | 164.14 (8) |
|  |  |  | Cu1—Cl3—Cu1^ⅳ^ | 162.95 (10) |

Symmetry code(s):

(i) x,-y,-1/2+z; (ⅱ) x,1-y,1/2+z; (ⅲ) x,-y,1/2+z; (ⅳ) x,1-y,-1/2+z.
